# *Rhyzobius lophanthae* Behavior is Influenced by Cycad Plant Age, Providing Odor Samples in a Y-tube Olfactometer

**DOI:** 10.3390/insects9040194

**Published:** 2018-12-18

**Authors:** Thomas E. Marler, Paris N. Marler

**Affiliations:** 1 College of Natural and Applied Sciences, University of Guam, UOG Station, Mangilao, Guam 96923, USA; 2 Centre for Sustainability PH, Inc., PENRO Road, Puerto Princesa, Palawan 5300, Philippines; pariscsph@gmail.com

**Keywords:** *Aulacaspis yasumatsui*, biocontrol, *Cycas micronesica*, olfactory signaling

## Abstract

The scale predator *Rhyzobius lophanthae* Blaisdell was introduced to Guam and Rota to control invasive *Aulacaspis yasumatsui* Takagi armored scale infestations on the native *Cycas micronesica* K.D. Hill populations. The predator demonstrated a clear preference for *A. yasumatsui* infesting adult plants, resulting in 100% seedling mortality due to the lack of a biocontrol of the scale on seedlings. A Y-tube olfactometer was employed to determine if scale-infested seedling leaves were less attractive to *R. lophanthae* than scale-infested mature tree leaves. Five paired combinations of seedling versus mature tree leaves were used. The *R. lophanthae* adults navigated toward scale-infested and un-infested leaves of adults and seedlings when paired with an empty chamber. However, a clear preference for adult leaves occurred when paired with seedling leaves. The results were unambiguous in charcoal-filtered air, intermediate in unfiltered air from an open laboratory, and most ambiguous when conducted with unfiltered in-situ air. The number of predators that did not make a choice was greatest for in-situ air and least for charcoal-filtered air. These results indicated that the substrate used in olfactometry influenced the results, and interpretations of charcoal-filtered air assays should be made with caution. Volatile chemical cues are involved in *R. lophanthae* preferring *A. yasumatsui* located on *C. micronesica* adults when infested adult and seedling leaves are present.

## 1. Introduction

The armored scale *Aulacaspis yasumatsui* invaded Guam in 2003 then invaded Rota in 2007 [1]. The lethal infestations quickly established on the native cycad, *Cycas micronesica*. The plant population rapidly declined, with seedlings and small juveniles dying more rapidly than larger plants [2]. The scale predator *Rhyzobius lophanthae* was introduced in 2005, and its effectiveness in a biocontrol reduced the lethality of the ubiquitous *A. yasumatsui* [1]. Two constraints on biocontrol were observed. First, the surfaces of cycad organs provided trichomes and other structures under which *A. yasumatsui* could hide from the predator [3,4]. Second, a clear stratification behavior occurred among the *R. lophanthae* predators, whereby they neglected scale-infested leaves located in the lowest forest strata [5,6]. 

These phenomena created insular tri-trophic relationships where *A. yasumatsui* remnant populations could avoid predation. These remnant populations could elicit irruptions any time a localized decline in the *R. lophanthae* population occurred [7]. The extent of plant mortality due to the lack of a biocontrol generated the Red-listing of *C. micronesica* by the International Union for Conservation of Nature [8] and listing under the United States Endangered Species Act [9]. More research to determine the drivers of these predator behaviors was needed to inform biological control management decisions designed to conserve the cycad population more effectively.

Insect search behavior is mediated by many cues. The countless sensory inputs and odor sources have been studied extensively for insect herbivores and predators. For our case study, we studied what mechanisms might cause a scale predator to avoid a readily available food resource just because it is located on a seedling leaf rather than a mature tree leaf. To address this topic, we employed olfactometry to determine if volatile chemical cues from leaves of different aged plants would influence the walking direction responses of *R. lophanthae*.

## 2. Materials and Methods

### 2.1. Preliminary Trials

We used a linear olfactometer to observe the search behavior of *R. lophanthae* adults when given contrasting odor sources. A plastic tube with a 15 mm inner diameter was used, and the olfactometer was constructed by cutting a hole at the midpoint of the tube into which the beetles could be introduced. The beetles were offered odor choices on each end of the horizontally oriented 30 cm tube using 3.78 L polyacetate bags containing leaf samples. Compressed air was regulated to 0.3 L·min^−1^ and directed into the sample bags, and the headspace air from the sample chambers passed into the openings of the olfactometer. The paired choices included scale-infested adult plant leaves versus empty, scale-infested seedling plant leaves versus empty, and scale-infested adult versus scale-infested seedling leaves. The mature leaf area was standardized by using a short section of the adult leaf rachis that contained a leaflet area that matched that of the corresponding seedling leaf area. Unsexed adult beetles were collected from in-situ forest settings and transported to the experimental site in a cardboard box. Beetles were loaded individually into the hole, and the walking direction choices were recorded until 10 beetles had selected one of the two sources. The assays were conducted from 900 h to 1200 h. We recorded no choice responses after a beetle failed to make a binary choice within 3 min. No beetle was used two times to ensure that every animal was naïve.

### 2.2. Experimental Trials

Glass Y-tubes with a 13 mm inside diameter were used for binary choice tests in three settings. The main tube was 8 cm in length and the two arms were 5 cm in length. Sample chambers were created with 3.78 L polyacetate bags and affixed to the ends of the two arms. Compressed air was regulated to 0.3 L·min^−1^ and directed into the sample bags. The paired choices included scale-infested adult plant leaves versus empty, scale-infested seedling plant leaves versus empty, scale-infested adult versus scale-infested seedling leaves, un-infested adult plant leaves versus empty, and un-infested seedling leaves versus empty. Leaf samples were collected in-situ from naturally infested plants, and uninfested leaves were collected from plants that did not exhibit noticeable scale infestations. The infested leaflets were restricted to leaves with 100% infestation on the adaxial surfaces and no infestation on the abaxial surfaces. This infestation pattern is widespread. The apical region of each seedling leaf rachis was clipped to ensure that every leaf sample had a rachis wound on both ends of the leaf section. All assays were conducted from 900 h until 1200 h to standardize the time of day among all trials.

The tests were first conducted with standard protocols of charcoal-filtered air under ambient conditions of an open-air laboratory at the University of Guam. The filter contained 9 mL of charcoal substrate. The assays were then conducted in the same laboratory using unfiltered air. The site was surrounded by secondary forest and urban landscape specimens. Cultivated *Cycas micronesica* and *Cycas revoluta* Thunb. plants were located in close proximity to the test location. The assays were conducted a third time in an in-situ forest setting, with scale-infested *C. micronesica* plants surrounding the experimental site.

Twenty unsexed adult beetles were loaded individually into the horizontally oriented olfactometer for each paired bioassay for each trial. We conducted 10 trials for each of the prescribed bioassays. The beetles were collected in-situ within 2 h of the bioassays and stored in a dark cardboard box until use. For the in-situ assays, the beetles were collected from a different habitat to ensure that inexperienced beetles were used. A decision toward one of the two choices was recorded when the beetle walked past a decision line at 3 cm from the junction. The beetles were not allowed to enter the chambers to ensure that no semiochemicals were added to the odor chambers. When the search choice was recorded, the chambers were immediately removed from the Y-tubes and the beetle was removed through the main entry tube. If the choice lines were not crossed within 3 min, the beetle was recorded as no choice. Each Y-tube was cleaned with ethanol and then water, and allowed to dry before being reused. The odor chambers were not replaced after each assay, and were affixed to sequential clean Y-tubes until 20 choices had been recorded for one of the three choices (the two odor chamber choices and no choice). The odor chamber sides were changed periodically to avoid position effects. Beetles were released following use and not re-used to ensure that every beetle was naïve.

### 2.3. Statistics

The non-parametric Kruskal-Wallis *H* test [10] was used to determine significance among the three choices for each paired bioassay. Post-hoc means separations were conducted using Bonferroni corrections of *p* values.

## 3. Results

### 3.1. Preliminary Trials

The number of beetles that the infested mature leaf chamber attracted was 10 and was 5.0-fold greater than the number attracted to the paired empty chamber (two). The number of beetles that the infested seedling leaf chamber attracted was 10 and was 1.6-fold greater than the number attracted to the paired empty chamber (six). The number of beetles that the infested mature leaf chamber attracted was 10 and was 2.0-fold greater than the number attracted to the paired infested seedling leaf chamber (five). In these preliminary tests, two to four of the beetles failed to make a choice toward either chamber. These preliminary trials revealed the utility of an olfactometer system for observing *R. lophanthae* search behavior using scale-infested *C. micronesica* leaves.

### 3.2. Experimental Trials

The use of a charcoal-filtered substrate generated clear choice responses in our bioassays. When paired with an empty chamber, 91% of the beetles chose the infested mature leaf chamber (Table 1). Similarly, when paired with an empty chamber, 93% of the beetles chose the infested seedling leaf chamber, 90% of the beetles chose the un-infested mature leaf chamber, and 90% of the beetles chose the un-infested seedling leaf chambers (Table 1). When the substrate was scrubbed of all background odor noise, the beetles were highly skilled at maneuvering toward *C. micronesica* leaf tissue, even if no scale food source was available. The preference for mature leaves was evident in the test with two leaf choices, where 89% of the beetles selected infested leaves from mature plants when the paired chamber contained infested leaves from seedling plants. Beetles that exhibited no preference were few in this substrate, ranging from 4% to 6% of the total number of beetles per assay (Table 1).

The use of an unfiltered substrate from an open-air laboratory generated similar trends, but the choice responses were less profound. Only 69% of the beetles chose the chamber with infested mature leaves when paired with an empty chamber (Table 2). While the differences were significant, a relatively high 31% of the beetles did not respond to the odor cues of the infested leaves. Similarly, 53% of the beetles chose the infested seedling leaf chamber, 53% of the beetles chose the un-infested mature leaf chamber, and 52% of the beetles chose the un-infested seedling leaf chamber when paired with an empty chamber (Table 2). Every response toward one of the leaf chambers was significantly greater than the corresponding responses to the empty chambers. The choice between infested mature leaves and infested seedling leaves indicated that 63% of the beetles selected infested leaves from mature plants. The number of beetles exhibiting no preference in these unfiltered bioassays was greater than for the charcoal-filtered bioassays, and ranged from 12% to 16% of the total number of beetles per paired test (Table 2).

The use of unfiltered air from an in-situ forest setting generated choice responses that were greatly different from both of the laboratory bioassays. Using unfiltered forest air, 61% of the beetles chose the infested mature leaf chamber over the empty chamber (Table 3). The beetle behavior when presented with infested seedling leaves was dissimilar to that of mature leaves, as only 49% of the beetles selected the infested seedling leaf chamber over the empty chamber. The un-infested leaf tests were similar for the two plant ages, as 39% of the beetles selected the un-infested mature leaf chamber and 38% of the beetles selected the un-infested seedling leaf chamber when paired with empty chambers (Table 3). When infested mature leaves were paired with infested seedling leaves, 49% of the beetles selected the mature leaves and 34% of the beetles selected the seedling leaves. In this forest setting, 26% of the beetles were unresponsive (Table 3). This was the only test setting where the number of beetles that exhibited no preference was not different from the number of beetles that selected the empty chamber for some of the paired odor tests.

We acknowledge that more binary combination choices, such as infested versus non-infested leaves, would greatly add to a better understanding of the tri-trophic dynamics in this system. Therefore, we urge caution in the full interpretation of our results.

## 4. Discussion

Insects navigate their environment in search of various resources using myriad behavioral processes and sensory inputs [11,12,13,14]. We have shown that olfactory cues are among the sensory inputs used in the walking search behavior of *R. lophanthae* scale predator adults to navigate toward a *A. yasumatsui* scale food source. An unambiguous preference for a scale food source located on mature *C. micronesica* leaves occurred. 

The muted ability of seedling leaves to employ this form of indirect plant defense by attracting the predator toward *A. yasumatsui* infestations may partly explain why *C. micronesica* seedling and juvenile plants died in epidemic rates following the invasion of Guam and Rota by *A. yasumatsui* [2]. The remaining live *C. micronesica* individuals on Guam and Rota are large mature specimens (personal observations).

Our ethology approach clearly distinguished plant volatile organic compounds as the mediating signals rather than *A. yasumatsui* semiochemicals, as the plant age used for the source leaf influenced the predator’s behavior. If *A. yasumatsui* semiochemicals were primarily responsible for the predator search behavior, the results would have been similar for the infested mature plant leaves and the infested seedling leaves.

We have also shown that search context exerted a profound influence on the predator’s behavior. In a filtered substrate absent of background olfactory noise, there was minimal difference in beetle behavior for the infested mature leaf and the infested seedling leaf when paired with empty chambers, and the number of beetles that failed to make an olfactometer choice was minimal (Table 1). In contrast, in the forest setting, more beetles responded to the infested mature leaves than the infested seedling leaves and all of the paired choice tests included numerous beetles that did not make a choice due to the complex olfactory noise in the substrate (Table 3).

The use of un-infested *C. micronesica* leaves in this olfactometer system also revealed the importance of the olfactory landscape. The number of beetles that the un-infested leaves from mature and seedling plants attracted was dissimilar. These un-infested leaf chambers exhibited only a 1.1-fold increase of attracted beetles above the empty chambers in the forest setting. In contrast, these un-infested leaf chambers exhibited a 17.2-fold increase of attracted beetles above the empty chambers for the charcoal-filtered substrate. Background olfactory variation in the air supply is not noise in the system that needs to be filtered to produce an artificial experimental environment, it is the system. No arthropod will experience the volatile cues that ethologists elect to study in the absence of this system, so caution is warranted when interpreting results from studies where background volatiles have been scrubbed. The importance of the volatile landscape for interpreting insect behavior has been discussed elsewhere [15,16,17,18].

Our results revealing an olfactometry preference for *A. yasumatsui* on mature *C. micronesica* leaves add to our previous reports indicating that *R. lophanthae* also avoids the lowest strata in the forest [5,6]. This vertical stratification behavior is a second factor enabling the ability of *A. yasumatsui* to lethally infest seedlings and small juvenile plants, despite the ubiquity of *R. lophanthae*. The size differential between the predator and pest also results in limitations of the biological control [3,4]. Our attempts to introduce parasitoid biological control species have been unsuccessful to date. Therefore, any improvement in our understanding of the limitations of the ubiquitous predator is needed. Ephemeral irruptions of the scale pest occur whenever localized predator populations decline. The many limitations of the predator biological control indicate that this spatiotemporal pattern of scale infestation will continue until a parasitoid can be successfully introduced. In the early years following the scale invasion, the predator was manually collected from high population sites and physically transported to new scale outbreak sites [19]. The limitations of this predator in the contemporary Guam and Rota tri-trophic systems indicate that sustained active management of the predator may improve *C. micronesica* conservation. For example, frequent scouting to locate ephemeral scale irruption locations would enable the physical transport of field-caught predators for reintroduction to these locations as a means of improving the biological control of this scale pest. 

## 5. Conclusions

Dual choice olfactometers were used to show that *R. lophanthae* predator adults navigated toward scale-infested and un-infested leaves of *C. micronesica* adults and seedlings when paired with an empty chamber. However, a clear preference for *A. yasumatsui* infesting adult leaves occurred when paired with infested seedling leaves. Volatile chemical cues are involved in *R. lophanthae* neglecting *A. yasumatsui* located on *C. micronesica* seedlings and preferring to feed on *A. yasumatsui* located on *C. micronesica* adult plants.

## Figures and Tables

**Table 1:** *Rhyzobius lophanthae* adult search responses to Y-tube olfactometer volatiles in charcoal-filtered air. Cumulative responses of 20 adults that made decisions in air that was charcoal-filtered. N = 10 trials, 200 adults total.

Assay Choice 1	Assay Choice 2	Assay Choice 3	Adjusted *H*	Significance
Infested mature 19b ^1^	Empty 1a	No preference 1a	24.7 ^2^	<0.0001
Infested seedling 19b	Empty 1a	No preference 1a	23.6	<0.0001
Infested mature 19b	Infested seedling 2a	No preference 1a	23.4	<0.0001
Un-infested mature 19b	Empty 1a	No preference 1a	23.1	<0.0001
Un-infested seedling 19b	Empty 1a	No preference 1a	23.1	<0.0001

^1^ Means followed by the same letter within each row are not different according to Bonferroni corrections for post-hoc comparisons. ^2^ Kruskal Wallis *H* statistic.

**Table 2:** *Rhyzobius lophanthae* adult search responses to Y-tube olfactometer volatiles in unfiltered laboratory air. Cumulative responses of 20 adults that made decisions in an open-air laboratory. N = 10 trials, 200 adults total.

Assay Choice 1	Assay Choice 2	Assay Choice 3	Adjusted *H*	Significance
Infested mature 16c ^1^	Empty 4b	No preference 3a	23.8 ^2^	<0.0001
Infested seedling 13c	Empty 7b	No preference 4a	26.1	<0.0001
Infested mature 15c	Infested seedling 6b	No preference 3a	29.4	<0.0001
Un-infested mature 12c	Empty 8b	No preference 3a	28.7	<0.0001
Un-infested seedling 12c	Empty 8b	No preference 3a	28.8	<0.0001

^1^ Means followed by the same letter within each row are not different according to Bonferroni corrections for post-hoc comparisons. ^2^ Kruskal Wallis *H* statistic.

**Table 3:** *Rhyzobius lophanthae* adult search responses to Y-tube olfactometer volatiles within in-situ settings. Cumulative responses of 20 adults that made decisions within in-situ forest. N = 10 trials, 200 adults total.

Assay Choice 1	Assay Choice 2	Assay Choice 3	Adjusted *H*	Significance
Infested mature 17c ^1^	Empty 3a	No preference 7b	25.3 ^2^	<0.0001
Infested seedling 13b	Empty 7a	No preference 7a	21.4	<0.0001
Infested mature 13b	Infested seedling 7a	No preference 7a	22.0	<0.0001
Un-infested mature 11c	Empty 9b	No preference 7a	18.6	<0.0001
Un-infested seedling 10c	Empty 10b	No preference 7a	17.4	<0.0001

^1^ Means followed by the same letter within each row are not different according to Bonferroni corrections for post-hoc comparisons. ^2^ Kruskal Wallis *H* statistic.
